# Impact of livestock production system on carcass and meat quality of Bouhachem Park goat kids

**DOI:** 10.5455/javar.2025.l936

**Published:** 2025-09-02

**Authors:** Zakaria Felloul, Sara Boukour, Yousra Zantar, Mohammed Ayadi, Abdelhay Arakrak, Mohammed Bakkali, Said Zantar, Amin Laglaoui

**Affiliations:** 1 Biotechnology & Biomolecular Engineering Research Team (ERBGB), Abdelmalek Essaadi University, Faculty of Science and Technology of Tangier, Tangier, Morocco; 2 National Institute of Agronomic Research (INRA), Regional Center of Agronomic Research of Tangier, Tangier, Morocco; † These two authors contributed equally to this work.

**Keywords:** Goat kids, livestock system, carcass traits, meat quality, fatty acid profile, sensory evaluation

## Abstract

**Objective:**

This study aimed to evaluate the impact of extensive and intensive production systems on the carcass traits and meat quality of northern Moroccan goat kids from Bouhachem Park, Morocco.

**Materials and Methods:**

Nine 6-month-old male goat kids were reared extensively on mountain pastures in the Chefchaouen region (Bouhachem Park) before slaughter. Body weights and linear measurements were recorded, and comprehensive analyses were conducted to evaluate the nutritional, technological, and sensory characteristics of their meat. The findings were then compared with data from nine intensively reared male goat kids.

**Results:**

Extensively reared goat kids demonstrated significantly superior growth performance and carcass characteristics (*p *< 0.05). Their meat exhibited lower fat content (2.24/100 gm *vs*. 5.23/100 gm) and reduced cholesterol levels (28.87 mg/gm *vs*. 40.07 mg/gm) compared to meat from intensively reared goat kids. Additionally, the fatty acid profile was more favorable, characterized by a lower proportion of saturated fatty acids (45.77% *vs.* 47.48%), a higher polyunsaturated fatty acid content (16.85% *vs.* 8.72%), and an increased percentage of desirable fatty acids (72.84% *vs.* 70.52%). The semimembranosus muscle from extensively reared kids had significantly lower pH values (5.88 *vs.* 6.00). However, both outdoor- and indoor-raised kids showed acceptable meat color redness and shear force values. Sensory panel evaluations indicated a clear preference for extensively reared goat meat in terms of color, flavor, herbaceous aroma, and overall acceptability.

**Conclusion:**

Goat kids from Bouhachem Park pastures demonstrated promising results in growth performance, carcass traits, and meat quality. Their meat represents a valuable dietary option, offering high nutritional value and a superior sensory profile.

## Introduction

According to the Food and Agriculture Organization, the global goat population reached approximately 1,145.49 million heads in 2022 [1]. Goat production systems differ widely due to ecological, economic, and cultural influences. The adaptability of goats to harsh environments and limited resources underscores their global importance in livestock production [2].

In northern Morocco, the goat population numbers around 788,000 head, with 70% reared in the mountainous regions of Chefchaouen and Tetouan provinces [3]. Bouhachem Natural Park, in northern Morocco’s Bouhachem Mountains, is a regional reserve within the southern Mediterranean ecological zone, where forest rangelands serve as crucial forage resources for domestic animals, especially goats [4]. The most abundant plant species include *Quercus ilex* (holm oak), *Quercus canariensis* (Zean oak), *Quercus suber* (cork oak), *Quercus coccifera* (kermes oak),* Arbutus unedo* (strawberry tree), *Genista scorpius* (thorny broom), *Cytisus scoparius* (Scotch broom), *Olea europaea* (European olive tree), *Pistacia lentiscus* (lentisk), *Calicotome villosa* (spiny broom), *Lavandula stoechas* (topped lavender), *Erica arborea* (tree heath), *Myrtus communis* (common myrtle), *Phillyrea media* (broad-leaved phillyrea), and *Rubus ulmifolius* (elm-leaf blackberry). *Quercus* species were identified as the most palatable plants for goat kids in Bouhachem Park during the summer and autumn [4]*.*

Goat meat is a rich protein source containing essential amino acids, yet it is low in intramuscular fat and cholesterol, making it a popular choice worldwide due to its lean composition and favorable nutritional profile [5]. Goat meat is generally characterized by its unique aroma, flavor, and textural properties. These attributes can vary significantly depending on the specific conditions under which the goats are raised and the methods used to process the meat [6]*.* Goats grazing on native pastures offer greater health benefits, as evidenced by higher levels of total omega-3 (n-3) polyunsaturated fatty acids (PUFAs) and a more favorable omega-6 (n-6) to n-3 PUFA ratio, alongside sensory attributes preferred by consumers [7]. Diet plays a significant role in determining the fatty acid composition of ruminants’ meat [8]. Feeds containing high levels of phenolic compounds, such as oak (*Quercus* spp.), inhibit fatty acid biohydrogenation in the rumen, thereby enhancing the deposition of unsaturated fatty acids (UFAs) into muscle tissue [3].

The quality of goat kid carcasses and meat is influenced by intrinsic factors (breed, age, sex, and slaughter weight) and extrinsic factors such as the production system, feed quantity and quality, pre-slaughter treatments, transport, and stress at slaughter, among others [9].

To date, no studies have evaluated the carcass and meat quality of goat kids raised in northern Morocco’s Bouhachem region. This study, therefore, investigates the effects of production systems and diets on carcass characteristics, growth performance, and the technological and nutritional quality of goat kid meat. The aim is to investigate the potential health benefits and sensory attributes of this meat for consumers.

## Materials and Methods

### Ethical approval

All housing, feeding, and handling procedures were approved by the National Institute for Agronomic Research—Regional Center of Tangier (approval number: 935/INRA/DGRHF/DC/3). Ethical guidelines were strictly followed, and all possible measures were taken to minimize animal suffering.

### Animals and diets

This study was conducted at the National Institute of Agricultural Research (INRA) in Tangier, Morocco (35°43’51.0”N, 5°52’56.7”W, 11 m above sea level). Nine 6-month-old male goat kids of a local indigenous breed, weaned at 3 months of age and with initial weights ranging from 16.82 ± 2.2 kg to 20.18 ± 1.32 kg, were slaughtered to evaluate carcass and meat quality. The kids were reared under a pastoral system, grazing freely in the pastures of Bouhachem Park. A 6-month-old kid consumes an average of 570 gm dry matter (DM) per day [4].

For comparison, nine intensively reared kids, also 6 months old and weaned at 3 months, were housed at the INRA experimental farm. Their standard diet consisted primarily of coarse feed, including oat hay (5 kg per day per lot), supplemented with barley grains (2 kg) and fava bean grains (3 kg). Feed amounts were adjusted based on weight progression, following the INRA feeding system [10] with negligible food refusal. The experimental period lasted 90 days (summer) for both extensive and intensive goat kid production systems.

### Slaughter procedure and meat collection

The kids were fasted for 12 h with free access to water and weighed before slaughter (live weight before slaughter, LWBS). Slaughter was conducted following standard Halal procedures, with particular care taken to minimize stress. The head, feet, skin, thoracic organs (heart and lungs), and viscera (including the full and empty digestive tract, liver, and kidneys) were weighed. Hot empty carcasses (HEC) were weighed, kept at room temperature (22°C ± 3°C) for 6 h to prevent cold shortening, and then chilled at 4°C for 24 h post-mortem. The dressing percentage (DP) was calculated using the following formula [11]:


$$\mathrm{DP}\;=\;\frac{\mathrm{HEC}}{\mathrm{LWBS}}\;\times \;100$$

At 24 h post-mortem, carcasses were split longitudinally along the vertebral column, and each half was subdivided into six primal cuts. Samples of the *longissimus dorsi* (LD) and *semimembranosus* (SM) muscles were excised from each carcass for meat quality assessment. All samples and cuts were labeled, vacuum-sealed, and stored at −25°C until analysis, with storage duration not exceeding 30 days.

### Technological analysis

Before packaging, the pH of the two muscle types (LD and SM) was measured. Initial and ultimate pH (pHu) values (after 24 h) were recorded using a portable meat pH meter (HANNA pH Meter HI99163). Meat color was assessed 24 h post-mortem on a freshly cut LD muscle surface using the CIE color system with a Konica Minolta CR-400 colorimeter [12]. Calibration was performed using a white surface (Illuminant D65: *Y* = 86.3; *X* = 0.3180; *y* = 0.3350). The CIE parameters included L* (lightness), a* (redness), and b* (yellowness). The hue angle (H°) and chroma (C) were calculated using the following formulas:


$$C\;=\;\sqrt{a^{*2}\;+\;b^{*2}}\;;\;\;\;H^{o}\;=\;\tan^{-1}\;(\frac{b^{*}}{a^{*}}\mathrm{).}$$

The remaining technological analyses were conducted on the SM muscle. Water-holding capacity (WHC) was determined by applying a 2.25 kg weight to a meat sample placed between two filter papers for 5 min. The WHC percentage was calculated using the following formula [13]:


$$\mathrm{WHC}\%\;=\;\left(\frac{\mathrm{meat}\;\mathrm{initial}\;\mathrm{weight}\;-\;\mathrm{Pressed}\;\mathrm{meat}\;\mathrm{weight}}{\mathrm{meat}\;\mathrm{initial}\;\mathrm{weight}}\right)\;\times \;100$$

For the cooking loss analysis, samples were weighed, sealed in plastic bags, and cooked in a water bath heated to 85°C for 45 min until they reached an internal temperature of 75°C. The bags were then drained and cooled at room temperature (25°C ± 3°C). The meat was reweighed, and the cooking loss percentage was calculated using the formula described by Liotta et al. [9]:


$$\mathrm{Cooking}\;\mathrm{loss}\%\;=\;\left(\frac{\mathrm{raw}\;\mathrm{meat}\;\mathrm{weight}\;-\;\mathrm{cooked}\;\mathrm{meat}\;\mathrm{weight}}{\mathrm{raw}\;\mathrm{meat}\;\mathrm{weight}}\right)\;\times \;100$$

Three subsamples (1 × 1 × 3 cm) were collected from the cooked meat, and shear force was measured using a Warner–Bratzler shear force apparatus connected to a Lloyd Instruments/Ametek TA1 texture analyzer. The texture analyzer was equipped with a 500 N load cell and set to a crosshead speed of 200 mm/min [14].

### Nutritional analysis

To determine the moisture content, a 5-gm sample was mixed with purified sand (treated with 3 N hydrochloric acid and 95% ethanol) and subsequently dried in an oven at 102°C ± 2°C for 16 h [15]. The ash percentage was obtained by carbonizing 5 gm of the sample with 1 ml of magnesium acetate solution (150 gm/l) in a Nabertherm incineration furnace at 550°C for 4 h [16]. The protein percentage was determined using the Kjeldahl method [17].

The extraction and quantification of intramuscular fat were performed following the method described by Chaosap et al. [18]. A 2:1 mixture of trichloromethane (99%–99.4% GC grade, Sigma-Aldrich) and methanol (99% GC grade, SupraSolv) was used for extraction. Fatty acid methyl esters (FAMEs) were analyzed using gas chromatography with flame ionization detection. FAMEs were prepared through an acid-catalyzed method, as described by Aldai et al. [19], using a boron trifluoride-methanol solution (1.3 M, for GC derivatization; Sigma-Aldrich). Column and GC conditions were set according to the method of Liotta et al. [9]. FAMEs were identified by comparison with a standard analytical mixture comprising 37 fatty acids (C4–C24; FAME standard, Sigma-Aldrich). Fatty acid classification and ratio calculations were performed using formulas referenced by Banskalieva et al. [20]. Lipid quality indices were calculated using formulas reported by Ghaeni and Ghahfarokhi [21].

Cholesterol extraction was performed using a saponification method [22]. The hexane extract was evaporated under a nitrogen atmosphere, and cholesterol content was quantified using a colorimetric reagent composed of glacial acetic acid–FeSO₄–H₂SO₄ [23]. Absorbance was measured at 490 nm using a Thermo Scientific Evolution 201 spectrophotometer.

### Sensory analysis

Sensory evaluation was conducted in the sensory analysis laboratory at INRA, Tangier. A panel of 10 trained evaluators (five women and five men, aged 25–35 years) participated in the assessments. Samples were obtained from shoulder cuts of kid goats reared under extensive and intensive production systems. One day before sensory evaluation, the samples were thawed at 4°C for 24 h, wrapped in aluminum foil, and cooked in an oven at 180°C until the internal temperature reached 80°C. Subsequently, 10 subsamples (1 × 1 × 3 cm) were prepared and maintained at 60°C until serving. No salt or spices were added to the samples. Unsalted crackers and water were provided to panelists to cleanse their palates between assessments. Using an 8-point categorical scale, panelists evaluated appearance, tenderness, juiciness, odor intensity, flavor intensity, flavor quality, fat taste, residue, herbaceous aroma, and overall acceptability [24].

### Statistical analysis

Data were analyzed using the Statistical Analysis System, version 9.1 [25]. The effect of the production system was evaluated using one-way analysis of variance. Statistical significance was set at *p < *0.05.

## Results

### Growth performances and carcass characteristics

Table 1 presents the effects of the production system on linear measurements, carcass traits, and offal weights in goat kids. The results indicated that the farming system significantly influenced carcass and organ weights. Kids reared under an open-grazing system exhibited significantly higher LWBS and carcass weights (*p *< 0.01). In contrast, perirenal fat content was significantly lower in pasture-based systems (*p *< 0.05). Additionally, carcass length, thigh length, and thigh thickness were greater in kids raised under the extensive husbandry system.

**Table 1.** The effect of the production system on growth performance and carcass characteristics of goat kids.

Weights and measures	EB	IB	SEM	*p*-value
LWBS (kg)	18.32	15.06	0.53	**<0.001**
FC (kg)	14.46	11.62	0.33	**<0.001**
HEC (kg)	7.97	6.63	0.2	**0.004**
EDT (kg)	1.51	1.84	0.25	0.2858
DP (%)	43.57	44.04	3.38	0.6711
Skin (%)	6.96	4.67	0.53	**0.003**
Head (%)	7.25	6.5	0.05	**0.002**
Paws (%)	3.34	2.79	0.02	**<0.001**
Pluck (%)	4.02	4.28	0.02	0.068
Perirenal fat (gm)	41.02	49.34	5.45	**0.024**
K (cm)	47.02	45.56	2.04	0.1076
F (cm)	26.58	19.2	2.21	**0.003**
E (cm)	6.07	5.91	0.16	0.5093

DP = dressing percentage, E = thigh thickness, EB = extensive breeding, EDT = empty digestive tract, F = thigh length, FC = full carcass weight, HEC = hot empty carcass weight, IB = intensive breeding, K = carcass length, LWBS = live weight before slaughter.

*p*-value <0.05 shows a significant effect, and bold values denote statistically significant effects.

### Technological characteristics of meat

**Figure 1.**
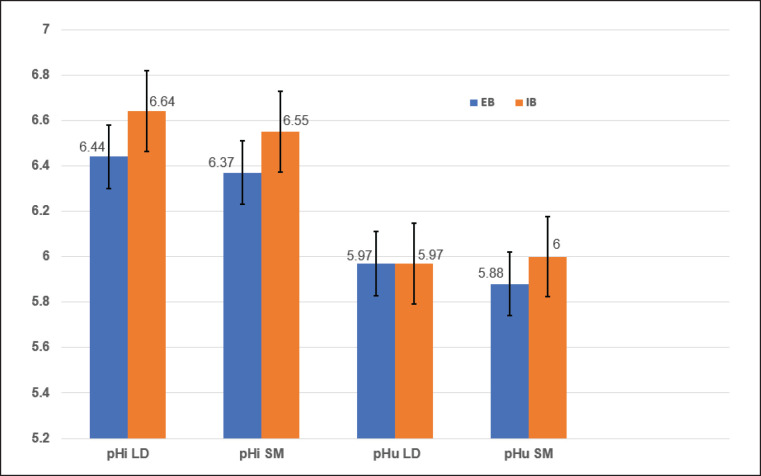
Breeding system effects on initial (pHi) and ultimate (pHu) pH values in goat kid meat.

Figure 1 illustrates the mean initial (pHi) and pHu values for LD and SM muscles. The SM muscle in outdoor-reared goat kids revealed a significantly lower pHu compared to other groups (*p *< 0.05). No differences were observed for the remaining pH values across muscles and rearing systems (*p *> 0.05).

Table 2 summarizes the technological characteristics of goat kid meat relative to the farming system. Extensively reared kids demonstrated significantly higher L* values (*p *< 0.05), lower WHC, and higher cooking loss percentages (*p *< 0.01). Raw meat from pastoral-reared kids showed significantly greater shear force values (*p* < 0.05); however, no significant differences were observed in shear force after cooking. Other characteristics did not differ significantly (*p *> 0.05).

### Nutritional analysis of meat

**Figure 2.**
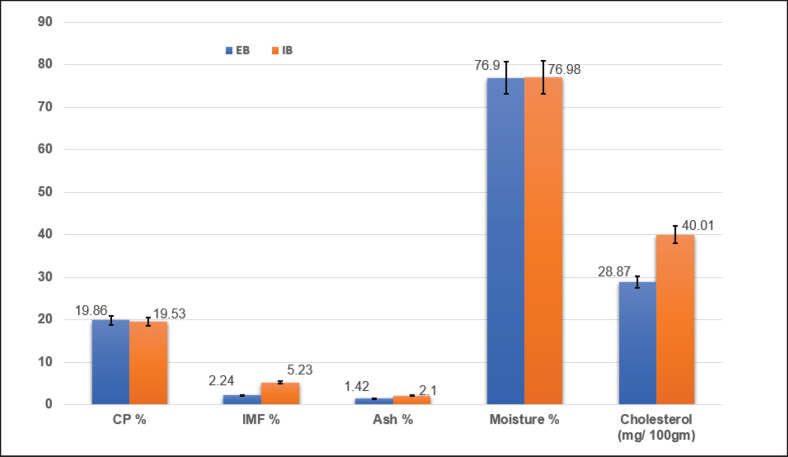
Chemical composition of outdoor- and indoor-raised goat kid meat samples.

Figure 2 presents the nutritional composition of goat kid meat from both production systems. The farming system had no significant effect on moisture or protein content. In contrast, ash, fat, and cholesterol levels were significantly lower in meat from extensively reared kids (*p *< 0.001).

Table 3 details fatty acid profiles and lipid indices in intramuscular fat across husbandry systems. Oleic (C18:1n9), palmitic (C16:0), stearic (C18:0), and linoleic (C18:2n6) acids were predominant. Meat from pastoral system kids presented significantly lower values of C14:1 (*p *= 0.002), C15, and C18:1n9c (*p *< 0.001), whereas the percentages of C18:2n6, C18:3n6, C18:3n3, and C22:6n3 were significantly higher (*p *< 0.01). Indoor-reared kid meat indicated the highest concentration of saturated fatty acids (SFAs) (*p *< 0.05). A significant variation was observed in mono UFA (MUFA) content, with confinement-reared kids exhibiting the highest values (*p *< 0.001). In contrast, the PUFA percentage was significantly greater in pasture-raised kids (*p *< 0.001). Free-range meat contained more desirable fatty acids (DFAs) and higher PUFA/SFA and UFA/SFA ratios (*p* < 0.05). Extensively reared meat had increased n-3 (*p* < 0.001), though the n-6/n-3 (n6/n3) ratio and atherogenicity index (AI) did not differ between systems. Higher values of C18+C18:1/C16 and thrombogenicity index (TI) were observed in meat from housed kids (*p *< 0.05).

**Table 2.** Influence of the livestock system on technological parameters (color parameters, shear force, WHC, and cooking loss) of goat kid meat.

Technological parameters	EB	IB	SEM	*p*-value
L* LD	40.01	45.53	3.86	**0.033**
a* LD	21.08	21.98	3.17	0.635
b* LD	5.02	6.27	2.26	0.359
C LD	21.77	22.89	3.43	0.585
H° LD	12.84	15.99	4.78	0.280
Shear force of raw meat (kgf/cm^2^)	11.46	8.77	3.24	**0.027**
Shear force cooked meat (kgf/cm^2^)	4.51	4.53	1.44	0.975
WHC%	26.60	29.30	1.66	**0.004**
Cooking loss%	44.60	40.68	2.49	**0.002**

a* = redness, b* = yellowness, C = chroma, EB = extensive breeding, H = hue angle, IB = intensive breeding., L* = lightness, LD = longissimus dorsi muscle, WHC = water holding capacity.

Bold values indicate statistically significant effects (*p* < 0.05).

### Sensory evaluation

Table 4 presents the outcomes of the sensory analysis conducted on goat kid meat from two livestock production systems. Sensory evaluation aimed to identify and quantify various sensory attributes and establish the meat’s sensory profile. Cooked meat samples were characterized by a light-brown color, tender texture, and pleasant aroma. Significant differences were observed between goat kids reared under extensive and intensive systems for most sensory attributes, except tenderness, juiciness, aroma, and overall acceptability. Panelists rated the appearance and flavor quality of meat from pasture-raised kids more favorably (*p* < 0.001), as well as herbaceous aroma and overall acceptability scores. Conversely, meat from confined goat kids expressed a more intense odor and a pronounced fatty taste (*p *< 0.001).

**Table 3.** Effect of breeding system on fatty acid profile, fatty acid groups, ratios, and indices in goat kid meat.

Fatty acid profile	EB	IB	SEM	*p*-value
C4	0.93	1.64	0.7	0.051
C12	0.16	0.12	0.01	0.38
C14	2.19	1.93	1.16	0.556
C14:1	0.13	0.33	0.01	**0.002**
C15	0.39	2.33	0.67	**<0.001**
C16	19.25	19.53	5.97	0.777
C16:1	1.19	1.39	0.31	0.382
C17	1.11	0.94	0.06	0.094
C17:1	0.82	0.76	0.18	0.392
C18	18.61	17.99	5.57	0.532
C18:1n9t	0.41	0.58	0.07	0.134
C18:1n9c	29.16	36.04	9.67	**<0.001**
C18:2n6t	0.38	0.09	0.01	**<0.001**
C18:2n6c	10.15	6.33	3.76	**<0.001**
C20:0	0.26	0.17	0.01	0.089
C18:3n6	2.17	0.43	0.11	**<0.001**
C20:1	0.45	0.23	0.02	**0.014**
C18:3n3	0.04	0.01	0	**0.009**
C21	0.08	0.83	0.12	**<0.001**
C20:2	0.49	0	0.01	**<0.001**
C22	0.56	0.34	0.04	**0.013**
C22:1n9	4.83	4.18	2.23	0.48
C20:3n3	0.08	0	0	**<0.001**
C24	1.64	1.09	0.51	0.077
C20:5n3	0.01	0	0	0.157
C24 :1	0.24	0.16	0.01	0.172
C22:6n3	3.19	1.84	0.32	**<0.001**
FAs groups				
SFA	45.77	47.48	3.73	**0.041**
MUFA	37.37	43.80	4.32	**<0.001**
PUFA	16.85	8.72	7.10	**<0.001**
UFA	54.23	52.52	3.74	**0.041**
DFA	72.84	70.52	6.81	**0.04**
n-6	12.76	6.86	4.94	**<0.001**
n-3	3.32	1.85	0.33	**<0.001**
Ratios				
PUFA/SFA	0.37	0.18	0.01	**<0.001**
UFA/SFA	1.19	1.10	0.01	**0.044**
n6:n3	3.84	4.1	1.25	0.577
Indexes				
C18+C18:1/C16	2.53	2.83	0.12	**0.044**
AI	0.52	0.52	0.02	0.962
TI	1.14	1.27	0.02	**0.038**

AI = atherogenicity index, DFA = desirable fatty acids, EB = extensive breeding, IB = intensive breeding., MUFA = mono-unsaturated fatty acids, n-3 = Oméga3, n-6 = Oméga6, PUFA = polyunsaturated fatty acids, SFA = saturated fatty acids, TI = thrombogenic index, UFA = unsaturated fatty acids.

Bold values indicate statistically significant effects (*p* < 0.05).

**Table 4.** Sensory test results of goat kid meat from extensive and intensive systems.

Sensorial attributes	EB	IB	SEM	*p*-value
Appearance	4.19	3.14	0.74	**< 0.001**
Odor intensity	4.36	5.75	0.70	**< 0.001**
Tenderness	6.26	6.45	0.30	0.085
Juiciness	4.2	4.21	0.70	0.957
Flavor intensity	4.49	5.05	0.71	**0.001**
Flavor quality	6.10	4.02	0.41	**< 0.001**
Fat taste	3.07	5.74	0.42	**< 0.001**
Residue	2.56	2.91	0.43	**0.009**
Herbaceous aroma	6.04	5.71	0.81	0.064
Overall acceptability	6.72	5.98	0.52	0.101

EB = extensive breeding, IB = intensive breeding.

Bold values indicate statistically significant effects (*p* < 0.05).

## Discussion

### Carcass quality

LWBS was significantly higher in kid goats raised under an extensive system, likely due to differences in feed resources between the two production systems [24]. The elevated LWBS values may link to the observed higher carcass weights. The reduced perirenal fat content and greater thigh length could be attributed to increased physical activity from grazing in pastures throughout the day [26]. However, studies such as Marques et al. [27] report that goats grazing on native pastures typically exhibit slower growth rates and lower carcass yields compared to concentrate-fed animals. This contrasts with our findings, which may arise from regional variations in the nutritional composition of pasture plants, which could affect growth outcomes.

Goat kids reared in a confinement system presented a higher percentage of empty digestive tract weight. This is likely attributed to their adaptation to diets rich in roughage and concentrates, which promote greater development of the gastrointestinal tract (stomach and intestines) [28]. The DP of goat carcasses in this study aligns with the typical range reported for goats (50%–55%) [29]. This value is generally lower than that of sheep, due to reduced fat deposition in goat carcasses [30]. This percentage is influenced by various factors, including the goat’s genotype, fatness, diet, and pre-slaughter conditions. Understanding these factors is essential for producers aiming to optimize meat yield and profitability [31].

### Technological quality of meat

The assessment of technological quality aims to evaluate the meat’s capacity to maintain freshness throughout its shelf life. The pHu of kid goat meat in this study fell within the typical values cited by Corazzin et al. [32] (5.8–6.2). However, Yaacoub [33] found higher values (6.29). The difference is due to the inherently excitable nature of goats, which renders them more susceptible to emotional stress [3]. Stress before slaughter causes hormonal changes that use up energy stored in muscles, which slows down the process that makes the meat more acidic. A pH above 6.0 results in darker meat due to reduced protein denaturation and higher water retention [34].

Consumers generally prefer pale or pink meats [35]. However, in this study, free-range goat kids exhibited lower L* values, which reflect their reduced fat content [32]. In the present study, the goat kids were fed a diet containing various oak species, which are natural sources of polyphenols. Similarly, research on tea catechins (a class of polyphenols) demonstrated improved meat color stability in goats by inhibiting lipid oxidation and minimizing drip loss [36]. This suggests that polyphenol-rich diets, such as those containing oak species, may influence meat color metrics through analogous antioxidant mechanisms.

Tenderness, a key attribute influencing consumer preference, is affected by factors such as age, breed, and gender [37]. According to Belew et al. [38], cooked meat with a shear force exceeding 4.6 kg is classified as “tough.” In our study, the tenderness of kid meat was below this specified threshold, thereby classifying it as tender meat. This result contrasts with findings by Liotta et al. [9], who reported higher shear force values (5.04–5.55 kgf/cm^2^) for cooked meat from extensively raised male goat kids. No significant difference in shear force values was observed between goat kids reared under extensive and intensive systems in the present study. However, the significantly higher shear force values in raw meat are frequently linked to its low-fat content, collagen composition, and muscle structure [39].

The WHC of meat is a key indicator of juiciness and significantly influences its functional properties and processing characteristics [40]. In this study, the WHC percentage of kid goat meat aligned with the typical values (~30%) reported for young animals [41]. Sariçicek [42] mentioned a higher percentage for Angora male goat kids grazing on rangeland (32.22%–37.15%). The diet of goats significantly impacts the WHC of their meat [43].

Cooking losses are a significant factor because the water retained in the cooked product is a key contributor to its juiciness [44]. The cooking loss percentage in the present study ranged from 40% to 44%, higher than the percentages cited by Tüfekci and Olfaz [45] (28.15%) and by Kawęcka and Pasternak [29] (33.58%). In goat meat, cooking losses typically range around 35% or higher [46].

An inverse relationship was observed between pH and cooking loss, where higher pH values corresponded to a slight reduction in cooking loss. This is primarily due to the enhanced WHC of meat at higher pH levels, which reduces the amount of moisture lost during cooking [47,48]. This relationship was evident in our findings, as demonstrated by the comparison between the ultimate pH of the SM muscle and its corresponding WHC and cooking loss percentages.

### Nutritional quality of meat

The analysis of the nutritional composition of goat meat is a critical factor in assessing its quality and highlighting its nutritional value for consumers. In the present study, the protein content of goat kids’ meat was lower than that mentioned by Migdal et al. [49] for male goat kids (20.19% ± 1.01%), but fell within the range specified by Webb et al. for protein content in goat kids is about 170–292 gm/kg. A pasture-based feeding diet results in higher protein and lower fat concentrations in the meat compared to a concentrate-based diet [50].

The ash content of indoor-reared kid goats’ meat was higher, likely due to their diet, which includes concentrate feeds rich in minerals. These feeds evidently contribute to the increased mineral content observed in their meat [51]. In the same setting, a study conducted on Kacang kid goat meat raised intensively revealed an average value of 1.87 ± 0.18 [52]. In another investigation, Sariçicek [42] found higher ash content (3.62% ± 0.27%) in Angora goat kids grazing on rangeland. Red meat is regarded as a vital source of essential minerals for human nutrition, including iron, selenium, and zinc [53].

The moisture content of kid goats’ meat in this study was close to that found by Tomović et al. [54], with an average of 76.42%, but higher than the average found by Choi et al. [37] for 6-month-old goat kid meat (73.61%). Pasture grazing significantly reduces intramuscular fat while increasing moisture content compared to stall feeding [55] This increase can be directly associated with the breeding system and age of goats [56].

Intramuscular fat affects key meat quality parameters, including juiciness, tenderness, and yellowness [57]. In this study, extensively raised goat kids demonstrated lower fat levels. van Wyk et al. [58] have found average values of 1.97% and 1.49% in Boer and Veld kid goat meats, respectively. Low fat content can result from the increased physical activity associated with outdoor rearing systems compared to the limited movement in confinement systems [7].

The cholesterol levels obtained in our study are comparable to those reported by Beserra et al. [59] for male Moxoto kid goats, which ranged from 20.5 mg/100 gm to 28.5 mg/100 gm. Several studies have demonstrated higher values of cholesterol in goat kid meat from intensive fattening [49,60,61]. The significant difference in fat and cholesterol content between the two production systems can be attributed to both feed composition and physical activity levels. Meat from extensively reared goat kids is a healthier option for individuals with high low-density lipoproteins (LDLs) cholesterol due to its lean composition, lower SFAs, and reduced cholesterol levels. Goat meat further improves blood lipid profiles by increasing HDL (“good” cholesterol) and lowering total cholesterol, atherogenic index, and LDL [62].

The fatty acid profile in meat fats plays a significant role in determining sensory properties and nutritional quality, thus affecting consumer acceptance and health [51]. Several studies have identified the major fatty acids in goat kid meat as oleic acid (C18:1n-9), palmitic acid (C16:0), and stearic acid (C18:0) [9,42,45,63], which are consistent with our findings. Studies such as Pinheiro et al. [12] revealed linoleic acid (C18:2n6) as a major fatty acid in kid goat meat from intensive fattening. El Otmani et al. [57] reported a higher concentration of C18:0 (17.7%) compared to C16:0 (11.2%) in intensively reared “Bni Arouss” goat kids.

Dietary variations can significantly influence the fatty acid composition of muscle lipids [64]. In this study, meat from the housed kid system contained higher levels of SFA. This finding can be attributed to the fact that ruminant diets rich in forage tend to increase the SFA content in meat [65]. SFAs are often linked to various health disorders, particularly due to specific fatty acids such as lauric acid (C12:0), myristic acid (C14:0), and C16:0, which have been associated with an increased risk of atherosclerosis in humans [66].

Data from this study showed that C18:1n9 had the highest proportion among the identified MUFAs, surpassing all other MUFAs. Rhee et al. [67] reported that C18:1n9 accounts for more than two-thirds of the MUFA in lipids extracted from the LD muscle.

In a study conducted by Tüfekci and Olfaz [45], Hair goat kids fattened under intensive conditions exhibited a MUFA content of 67.30%, higher than that of those raised under semi-intensive (66.22%) and extensive systems (65.72%). In contrast, the MUFA percentages in the meat of indoor-reared kid goats in this study were significantly lower.

Conversely, meat from the open grazing system in the present study expressed higher values of PUFAs, likely due to the diverse plant species available in the pastures of Bouhachem Park. n-3 fatty acids are well known for their health benefits, including the prevention of cardiovascular diseases and their anti-inflammatory properties [7].

The meat of goat kids in this study demonstrated a favorable lipid profile with higher percentages of UFAs, ranging from 52.52% to 54.23%. Several studies have shown that UFA still predominates in goat meat, with concentrations averaging around 50% [68–71]. In the rumen, dietary UFAs are biohydrogenated by microorganisms, primarily resulting in the formation of C18:0 as the final product [32].

The PUFA/SFA ratio is widely recognized as an important dietary indicator of meat quality due to its impact on cardiovascular and metabolic health [72]. In this study, the PUFA/SFA ratio for pasture-raised kid goats was 0.37. According to Santos et al. [35], this ratio should exceed 0.4 to ensure balanced nutritional value and support the prevention of coronary heart disease.

The health implications of fatty acid composition extend beyond individual PUFA or SFA values. The three predominant fatty acids in muscle lipids have distinct effects on human cholesterol levels [73]. C16:0 is associated with an increase in blood cholesterol, while C18:0 has a neutral effect, and C18:1 is known to reduce blood cholesterol levels [74]. Consequently, Banskalieva et al. [20] proposed that the C18:0 + C18:1/C16 ratio serves as a valuable indicator for assessing the health impact of lipid profiles in meat. The mean value observed in this study (2.53 ± 0.3) falls within the range reported by Ding et al. (1.37–3.67) [75].

The DFA content is another critical factor in evaluating the nutritional value of meat. Goat kids in our study revealed higher percentages of DFA (72.84% and 70.52%). Mushi and Eik [76] reported that the total DFA proportion in goat meat ranges from 61% to 80%.

The balance of n-3 and n-6 fatty acids is also crucial for human health. The n-3 and n-6 fatty acid percentages in this study closely matched the values reported by Yalcintan et al. [63], with n-3 at 2.95% and n-6 at 8.63%. The n-6/n-3 ratio for free-grazing kid goats in this study was 3.84, which remains below the threshold of 4. This ratio is considered a favorable factor for the prevention of cardiovascular disease [35]. Diet composition, particularly the intake of polyphenol-rich plants, further influences meat lipid quality. Oak leaves, for example, are among the richest sources of polyphenols [77]. Studies have shown that polyphenol consumption in ruminants improves the fatty acid profile, resulting in healthier lipid composition [78]. This effect may partially explain the enhanced nutritional profile of kid goat meat in this study.

Fatty acids with AI action primarily belong to the n-6 PUFA class, while those contributing to TI effects are associated with the n-3 PUFA class [9]. The AI and TI values for grass-fed kid goats in this study were similar to those in braised beef liver, which is considered good for preventing blood clots (AI = 0.41 and TI = 0.82).

The research shows that goat meat from free-range farms has important health benefits, including lower fat and cholesterol levels and a better mix of fatty acids, with more polyunsaturated and good fatty acids. These attributes present valuable opportunities for marketing initiatives targeting health-aware consumers, particularly by highlighting the product’s cardiovascular advantages and exceptional nutritional profile. Additionally, the results provide scientific support for nutritional labeling, enabling producers to communicate the health advantages of free-range goat meat. Such findings could enhance market reach and consumer confidence in product quality.

### Sensory quality

The meat from the extensive system of kid goats in Bouhachem Park was well appreciated by the sensory panelists. Scores for appearance, flavor quality, herbaceous aroma, and overall quality were high, with a low perception of fatty taste and residue. Juiciness received a moderate rating, which aligns with the observed cooking loss percentages (44%). The low intensity of fatty taste was further confirmed through experimental analysis. The meat exhibited a refined flavor profile with a distinct herbaceous aroma, attributed to the richness of plant species in Bouhachem Park. Research has shown that a higher proportion of MUFAs, particularly oleic acid, positively influences sensory palatability [79], which aligns with the findings of this study. The higher intensity of the odor in cooked meat from confined goat kids is a result of several factors, including diet, intramuscular fat content, and rearing practices [80,81].

Opportunities for enhancing our study include expanding its geographic scope, as it focuses primarily on goat kids from Bouhachem Park. Evaluating goat kids from additional regions and parks within Chefchaouen city would enhance the generalizability of the findings. Furthermore, the variety of meat samples analyzed was limited; thus, future studies should assess multiple cuts from the goat kid carcasses to provide comprehensive meat quality data. Future research should systematically explore environmental factors such as seasonal variations and diverse livestock management practices.

## Conclusion

This study demonstrates the significant influence of livestock production systems on the carcass, nutritional, and technological quality of kid goat meat. The superior nutritional profile of extensively reared goats from Bouhachem Park, including lower fat content, reduced cholesterol, a more favorable fatty acid composition, and outstanding sensory palatability, positions their meat as a promising dietary alternative for health-conscious consumers. Conducting longer-term feeding trials, consumer acceptance evaluations, and expanded nutritional profiling (e.g., amino acids, bioactive compounds, vitamins, and minerals) would further validate our results and support broader market applicability. The results hold potential for advancing sustainable agriculture in northern Morocco, promoting cost-effective, eco-conscious farming practices among Chefchaouen producers, and encouraging them to produce a niche local food derived from indigenous goat breeds, thus enhancing economic viability.

## List of abbreviations

a*, redness; AI, atherogenicity index; b*, yellowness; C, chroma; CP, crude protein; DFA, desirable fatty acids; DM, dry matter; DP, dressing percentage; E, thigh Thickness; EB, extensive breeding; EDT, empty digestive tract; F, thigh length; FAME, fatty acid methyl esters ; FC, full carcass weight; H, Hue angle; HDL, high-density lipoproteins; HEC, hot empty carcasses; IB, intensive breeding; IMF, intramuscular fat; INRA, National Institute of Agricultural Research; K, Carcass length; L*, lightness; LD, longissimus dorsi muscle; LDL, low-density lipoproteins; LWBS, live weight before slaughter; M, molarity; MUFA, mono-unsaturated fatty acids; n-3, Omega3; n-6, Omega6; pHi, initial pH after 45 min of slaughter; pHu, final pH after 24h of slaughter; PUFA, polyunsaturated fatty acids; SFA, saturated fatty acids; SM, semi membranous muscle; spp., species; TI, Thrombogenic index; UFA, unsaturated fatty acids; WHC, water holding capacity.
